# Statistical modeling and optimization of volatile fatty acids production by anaerobic digestion of municipal wastewater sludge

**DOI:** 10.1007/s11356-024-34091-2

**Published:** 2024-08-28

**Authors:** Jeniffer Gracia, Oscar Acevedo, Paola Acevedo, Jhessica Mosquera, Carlos Montenegro, Ivan Cabeza

**Affiliations:** 1 Universidad Distrital Francisco José de Caldas, 110221 Bogotá, Colombia; 2 Department of Chemical and Environmental Engineering, Faculty of Engineering, Universidad Nacional de Colombia, 111321 Bogotá, Colombia; 3 Faculty of Engineering, Design, and Innovation, Politécnico Grancolombiano, 110231 Bogotá, Colombia; 4 Universidad EAN, 111321 Bogotá, Colombia; 5 Energy, Materials and Environment Laboratory, Faculty of Engineering, Universidad de La Sabana, Autopista Norte, Campus Universitario Puente del Común, Km 7, 250001 Chía, Colombia

**Keywords:** Acidogenesis, Resource recovery, Acidogenic fermentation, Value-added products, Organic load, Primary sludge

## Abstract

Obtaining value-added products from renewable resources is limited by the lack of specific operating conditions optimized for the physico-chemical characteristics of the biomass and the desired end product. A mathematical model and statistical optimization were developed for the production of volatile fatty acids (VFAs) by anaerobic digestion of municipal sewage sludge. The experimental tests were carried out in triplicate and investigated a wide range of conditions: pH 9.5, 10.5, and 11.5; temperatures 25 °C, 35 °C, 45 °C, and 55 °C; primary sludge with organic loading (OL) of 10 and 14 g VS (volatile solids); and digested sludge with 4 and 6 g VS. Subsequently, a statistical search was performed to obtain optimal production conditions, then a statistical model of VFA production was developed and the optimal conditions were validated at pilot plant scale. The maximum VFA concentration predicted was 6975 mg COD (chemical oxygen demand)/L using primary sludge at 25 °C, initial OL of 14 g VS, and pH 10.5. The obtained third-degree model (*r*^2^ = 0.83) is a powerful tool for bioprocess scale-up, offering a promising avenue for sustainable waste management and biorefinery development.

**Supplementary Information:**

The online version contains supplementary material available at 10.1007/s11356-024-34091-2.

## Introduction

Bioconversion of waste streams has become an opportunity to accomplish environmental sustainability, where the production of value-added products is central. Consequently, renewable resources are highly demanded to develop next-generation technologies to produce fuels, chemicals, energy, and materials (Atasoy et al. 2018). In this context, anaerobic digestion (AD) has been presented as a processing technology with a significant role in the circular economy concept as a platform for valorizing heterogeneous wastes (Gonzalez et al. 2022).

Among the most typical AD products are methane, biohydrogen, and soluble biochemicals, mainly volatile fatty acids (VFAs), produced during the acidogenesis stage (Hunter et al. 2021; Mosquera et al. 2021; Ochoa et al. 2021a, b). VFAs are an intermediate AD product using waste streams such as primary sludge and organic waste as substrates (Sanchez-Ledesma et al. 2023; Gracia et al. 2020). AD comprises four stages: solubilization and hydrolysis of organic matter, acidification, acetogenesis, and methanogenesis. The acidogenic fermentation process occurs in the phases of acidogenesis and acetogenesis, and physical or chemical processes are commonly applied to improve the fermentation rate. In the next step, the fermenting bacteria convert the monomers into end products and VFAs, while microorganisms degrade the organic matter under anaerobic conditions. When VFAs are the target, finding conditions that promote yield and prevent methanogenesis is crucial, such as adjusting the pH below 6.0 or above 8.0.

Recent research has stated the need to change the current production methods of VFAs to biotechnologies with waste and wastewater. Research on recovering VFAs through AD has grown as VFAs have become increasingly in demand as an essential chemical component (Hernandez et al. 2018; Ochoa et al. 2021a, b; Atasoy et al. 2018). Additionally, VFAs have a high potential as a source of renewable carbon. They can be used in the food, pharmaceutical, and chemical industries and are considered a valuable feedstock for products such as bioplastics (Venkateswar Reddy et al. 2014), other biopolymers (Pérez-Zabaleta et al. 2021), biogas (Begum et al. 2018), biodiesel (Fortela et al. 2016), and biohydrogen (Sydney et al. 2018). In addition, the quest to reduce greenhouse gas emissions makes the development of new sustainable processes a viable and necessary alternative and must be based on renewability, degradability, and sustainability (Bhatia & Yang 2017). Previous research on optimizing the operating conditions and increasing the efficiency of biobased VFA production methods using renewable sources as substrate, based on the interactions of microbial communities (Pang et al. 2023; Valentino et al. 2021; Atasoy et al. 2018). Nevertheless, the analysis used as substrate primary sludge is scarce (Valentino et al. 2017; Pérez-Morales et al. 2021). VFA can be produced by mixing microbial culture and anaerobic fermentation of different substrates. The main variables that affect VFA production and their typical range of values are reaction time (4 to 15 days), pH (above 8 and under 6, depending on the substrate), and temperature, which has been studied from psychrophilic (5–30 °C) to thermophilic (+ 50 °C) conditions (Atasoy et al. 2018).

One of the leading waste streams generated worldwide is sludge from wastewater treatment plants (WWTPs). Sludge has been considered a substrate for VFA production (Zeng et al. 2023; Zhou et al. 2023; Pérez-Morales et al. 2021). According to Chen et al. (2017), anaerobic fermentation provides a new system for sewage sludge reduction and VFA production. In addition, it has been observed that the excessive content of organic compounds in sewage sludge provides a potential recovery of VFA. Therefore, it is necessary to obtain the optimal conditions to generate technology transfer, considering the potential industrial benefits of the availability of VFAs, particularly from acidogenic fermentation of WWTP sludge, and bearing in mind that research with this type of waste is still in its infancy. The new processes are expected to displace developments dependent on non-renewable carbon from using residual raw materials in the circular economy framework, as biobased VFA production is increasing its market demand due to potential applications and its cost-effective approach (Annamalai et al. 2020). The market value of VFAs depends on the specific VFA produced and is estimated between 400 and 2500 €/ton (Atasoy et al. 2018). Additionally, by taking advantage of the amounts of organic matter in acidogenic fermentation for VFA production, the environmental impacts generated during the disposal of WWT sludge are avoided.

The scope of this research goes from the laboratory scale, where the VFA yield is measured using analytical methods, then the construction of a statistical model of VFA production, followed by a statistical search for optimal production conditions and assessment of the statistical significance of this optimal point, and finally, the validation of these optimal conditions at the pilot plant scale. The statistical methodology used in this work has some peculiarities that were absent in previous works on optimizing VFA production. Finding optimal conditions for VFA production from batch experiments is frequently done by discretely selecting the optimal point (Moretto et al. 2019). However, a more continuous search has been performed either by multiple linear regression (Nabaterega et al. 2022) or, more often, by a two-degree polynomial regression from an optimal experimental design. The latter approach is response surface methodology (Hong & Haiyun 2010; Liu et al. 2018, 2021). In this work, we employed a polynomial regression methodology but needed a third-degree model to adjust the data satisfactorily. We also needed to extend the number of experimental points accordingly to explore the response function’s behavior better. The works mentioned above employing response surface methodology explored a relatively small range of the continuous variables, and they were frequently centered near a possible optimal point. In our case, the continuous independent variable range was much more comprehensive, and the response variable had such a rich behavior that a quadratic model could not fully represent it. Another peculiarity in our work is that we obtained 12 different polynomial regression models corresponding to different discrete pH conditions, sludge type, and organic load, effectively combining a discrete optimization strategy with a continuous one. This strategy required further analysis to ensure that the differences we found among the 12 discrete treatments were statistically significant.

## Materials and methods

The complete methodology consists of three main phases: (1) laboratory setup and experimental design, (2) statistical modeling and optimization of VFA production, and (3) pilot plant setup to validate the optimal production.

### Laboratory setup and experimental design

#### Inoculum and sewage sludge

A methanogenic granular sludge was used as inoculum from the stabilized anaerobic reactor of the industrial wastewater treatment plant of Alpina SA in Sopo, Cundinamarca (Colombia). A heat shock treatment was necessary for an acidogenic fermentation system; the inoculum was boiled for 30 min at 250 °C and then was cooled to room temperature before being added to each reactor (Rangel et al. 2020). VS (volatile solids) and TS (total solids) in the inoculum were 41.05 ± 0.04 and 49.83 ± 0.03 g/L, respectively.

The primary and digested domestic wastewater sludges used as substrates are from the “El Salitre” wastewater treatment plant (WWTP), the main plant of this type in Bogotá (Colombia). Primary sludge (PS) and digested sludge (DS) were used since the wastewater treatment plant did not have activated sludge due to the treatment technology used during the process. The average characteristics of each sludge are as follows: primary sludge, VS 39.16 ± 0.06 g/L, TS 52.35 ± 0.07 g/L, SCOD (soluble chemical oxygen demand) 31.6 g/L; digested sludge, VS 11.40 ± 0.08 g/L, TS 52.35 ± 0.12 g/L, SCOD 11.6 g/L. All the samples were taken in triplicate in 25 mL.

The inoculum was stored at 4 °C before the experiments, and the primary and the digested sludge were kept in a freezer at − 4 °C to avoid microbiological degradation before testing (Iglesias-Iglesias  2019).

#### Batch experiments to obtain volatile fatty acids

**Fig. 1:**
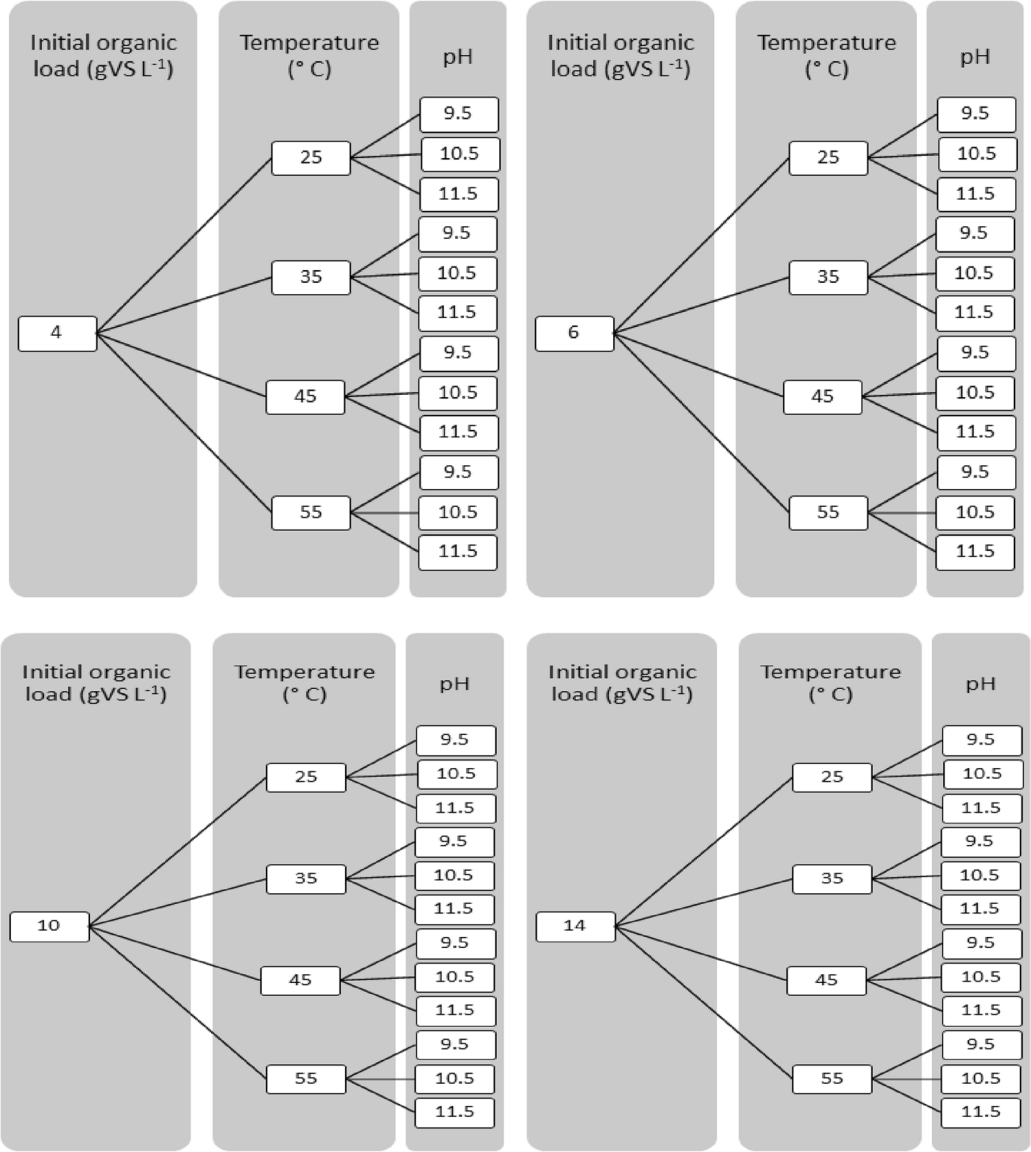
Experimental treatment conditions for the acidogenic fermentation of digested sludge and primary WWTP sludge. **a** Digested sludge, **b** primary sludge

The aim was to study the influence of different operating parameters on the production of VFA using El Salitre WWTP’s sludge by acidogenic fermentation and obtain the data needed to build the statistical model and carry out the process optimization. Figure 1 shows the developed experimental design, according to Zhang et al. (2019), considering sludge and inoculum characterization. The initial organic load (OL) of the reactors was set at two levels for digested sludge (6 gVS/L and 4 gVS/L) and (14 gVS/L and 10 gVS/L) for primary sludge. Also, the experiments include different levels of temperatures (25 °C, 35 °C, 45 °C, and 55 °C) and initial pH (9.5, 10.5, and 11.5). The tests used a buffer solution for each pH level to help control the reactors’ pH. The complete experimental design consisted of 48 combinations tested in triplicate, using 250-mL batch reactors and amber flasks with a working volume of 200 mL (Angelidaki et al. 2009). The triplicate repetition is used to estimate the variability of VFA production for each combination.

The substrate-to-inoculum (S/X) ratio was set to 1 to reduce inhibitory effects during fermentation (Rangel et al. 2020). The reactors contained the inoculum, the fixed organic load, the corresponding pH buffer solution, the NaOH solution to adjust the pH, and distilled water to reach the working volume. The reactors were hermetically closed and placed in a thermostatic bath to ensure mesophilic conditions. Then, every 3 days, three samples for each treatment combination were destroyed and analyzed to study the advance of the reactions. The experiment times at 25 °C, 35 °C, and 45 °C were 12 days when the reactors reported methane production (measured using BIOGAS 5000® Landtec). However, the time for the experiments at 55 °C reactors was only 9 days because their rate conversion was higher than for the other temperatures (Anantharaj et al. 2020).

In addition, each reactor had a volume displacement system to monitor the biogas yield, using a 0.5 N NaOH solution as a CO_2_ trap.

#### Analytical methods

The pH measurements were determined using an Edge pH meter model HI2002. TS, VS, and organic matter (OM) of the digested sludge and primary sludge samples were resolved by drying the samples at 105 ± 5 °C and subsequent calcination at 550 ± 10 °C according to 2540B APHA-SM and D3174 of the American Society for Testing and Materials (ASTM). The soluble chemical oxygen demand (SCOD) was measured using commercial vials from Hanna Instruments with a range of 0 to 150 mg/L (HI 93752). Total Kjeldahl nitrogen (TKN) was assessed according to ASTM D1426. The gas composition measurement (CO_2_, CH_4_, and O_2_%) was determined with the BIOGAS 5000® Landtec gas analyzer.

Volatile fatty acids (VFAs) and alkalinity (ALK) concentrations were measured according to standard methods (APHA, 2005). The best mixtures within the experimental design were subjected to a gas chromatographic (GC) quantification process, to determine the concentration of specific VFA, such as acetic, propionic, isobutyric, butyric, isovaleric, valeric, and isocaloric. Therefore, the samples were centrifuged twice for 15 min at 5500 rpm in a thermal centrifuge, Model Heraeus Megafuge 16. Next, the upper solution centrifuged was passed through 0.45 μm syringe filters until obtaining 0.9 mL. After this, 0.1 mL of a mixture of phosphoric acid and internal standard was added to the filter received. The final step was the GC/FID analysis of the mix. The chromatograph had the following specifications: Agilent 7890th gas chromatograph with CHEM STATION 32 data system, equipped with a capillary column (30 m × 25 mm × 0.25 μm), polar stationary phase of polyethylene glycol treated with nitro terephthalic acid, brand SGE Analytical Science (ref: BP21), automatic injector and FID detector. Chromatographic conditions were as follows: injection volume 1 μL, injector temperature 250 °C, pressure 19,414 psi, total flow 135.14 mL, purge flow at septum 3 mL/min, split injection mode 100:1, split flow 130.83 mL/min, average flow 13,083 mL/min, temperature programming 130 °C/min @ 6 °C/min up to 172 °C/min, carrier gas He, airflow 400 mL/min, H2 flow 30 mL/min, make-up flow N2 25 mL/min. The calculation of the VFA production yield results from the total VFA concentration in the effluent per gram of volatile solids (VS) fed (g COD/g VS), as can be seen in Eq. (1) (Garcia-Aguirre et al. 2017). This estimation allows the evaluation of the acidification potential of the primary and digested sludge.

1 $$\text{VFA yield }= (\text{VFA output}) / (\text{VS fed})$$

### Statistical methods

The following mathematical and statistical analyses were carried out to obtain the optimal VFA production from municipal wastewater treatment plant sludge:

#### Third-degree polynomial regression

Based on the data obtained in the laboratory phase, predictive regression models of VFA production as a function of time and temperature were devised. In these models, temperature is treated as a continuous variable, and its different values do not constitute separate discrete treatments. Since there is data for four different temperatures, 12 polynomial models (12 treatments) were obtained from the 48 original combinations of pH, temperature, sludge type, and load. The mathematical structure of the regression models is that of a two-variable third-degree polynomial. Consequently, the VFA production model for each treatment is defined by Eq. (2).

2 $$VFAs={\beta }_{0}+{\beta }_{1}t+{\beta }_{2}d+{\beta }_{3}{t}^{2}+{\beta }_{4}td+{\beta }_{5}{d}^{2}{+\beta }_{6}{t}^{3}+{\beta }_{7}{t}^{2}d+{\beta }_{8}t{d}^{2}+{\beta }_{9}{d}^{3}$$

where VFAs are the volatile fatty acids, $t$ is the temperature, and $d$ is the sludge retention time measured in days. The ${\beta }_{i}$ coefficients ultimately determine each regression model. An ordinary least squares algorithm estimated these coefficients from experimental data for each of the 12 treatments. The algorithm was implemented using Python, *sklearn* package version 0.24.2.

#### Optimization of volatile fatty acid production

Each of the 12 models found the time–temperature point of maximal VFA production. This optimal point was located by brute-force parameter sweeping with a resolution of 0.05 days and 0.05 °C. Analytical optimization methods like gradient descent were unnecessarily cumbersome because the parameter domain was restricted by methane production. However, the sweeping resolution is more than enough, considering experimental precision.

#### Bootstrapping

Bootstrapping was used to estimate the uncertainty in the optimal point of VFA production. First, assuming equal and normal variability in each experimental end, 300 sets of random observation points for each treatment were artificially sampled. Then, steps 2.2.1 and 2.2.2 were repeated for each group to obtain a simulated arbitrary optimal point. These random points are the starting points for steps 2.2.4 and 2.2.5, aiming to test the statistical significance of the results. Bootstrapping is an excellent way to estimate variability in complex algorithms described in steps 2.2.1 and 2.2.2, where an analytical estimation is either difficult or unfeasible.

##### ANOVA

The *F*-test analysis of variance was used to establish the statistical significance of the differences among the average optimal VFA production of each treatment. The average and variance were estimated by bootstrapping, and the ANOVA test was performed by setting the degrees of freedom for each treatment available in the polynomial regression. These steps aimed to demonstrate that the null hypothesis (that optimal averages can be assumed equal) should be rejected.

####  T-test 

This allowed us to determine whether there is a statistically significant difference between the two best-performing treatments: this test and the previous test.

#### Pareto analysis of t-values

A Pareto chart was made using the *t*-values of the ${\beta }_{i}$ polynomial coefficients. This analysis is vital to justify the third-degree polynomial model. If the statistical significance of third-degree coefficients (${\beta }_{6}$ to ${\beta }_{9}$) is high, this is an indication that a lower-degree polynomial could not capture well the overall behavior of data. On the other hand, we also checked that fourth-degree models did not perform much better despite requiring more parameters, i.e., leaving fewer degrees of freedom. In other words, we checked that our model choice is a good balance between simplicity and accuracy.

### Semi-continuous pilot plant experiment

**Fig. 2:**
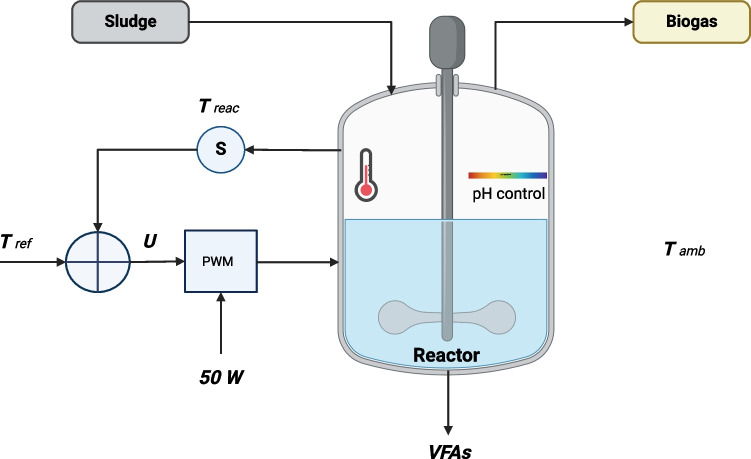
Schematic of the pilot plant system

Once the statistically optimal result for VFA production was obtained, it was replicated in a semi-continuous operation regime to validate the model, for which a pilot plant was set up with three automated reactors of 5-l capacity at the same pH and temperature conditions as the optimum and for 15 days, using primary sludge. Control measurements were carried out on the output variable VFAs on days 0, 3, 5, 7, 9, 11, 13, and 15. The quantification of the volume of biogas produced was carried out using RITTER flowmeters (MiligasCounter-RIGAMO software), which allows the measurement of total gas in real-time. The scheme is presented in Fig. 2.

## Results and discussion

### Volatile fatty acid production from digested sludge and primary sludge

The total VFA production was evaluated as concentration (mg COD/L) and yield (g COD/g VS). In general, combinations that use digested sludge (DS), despite already having consumed the VFA to produce biogas, can generate a good VFA yield, maximizing the production of value-added products. However, this depends on the WWTP's retention time (RT) and gas quality. The digester is a pretreatment to maximize the number of VFAs (Yuan et al. 2022).

**Fig. 3:**
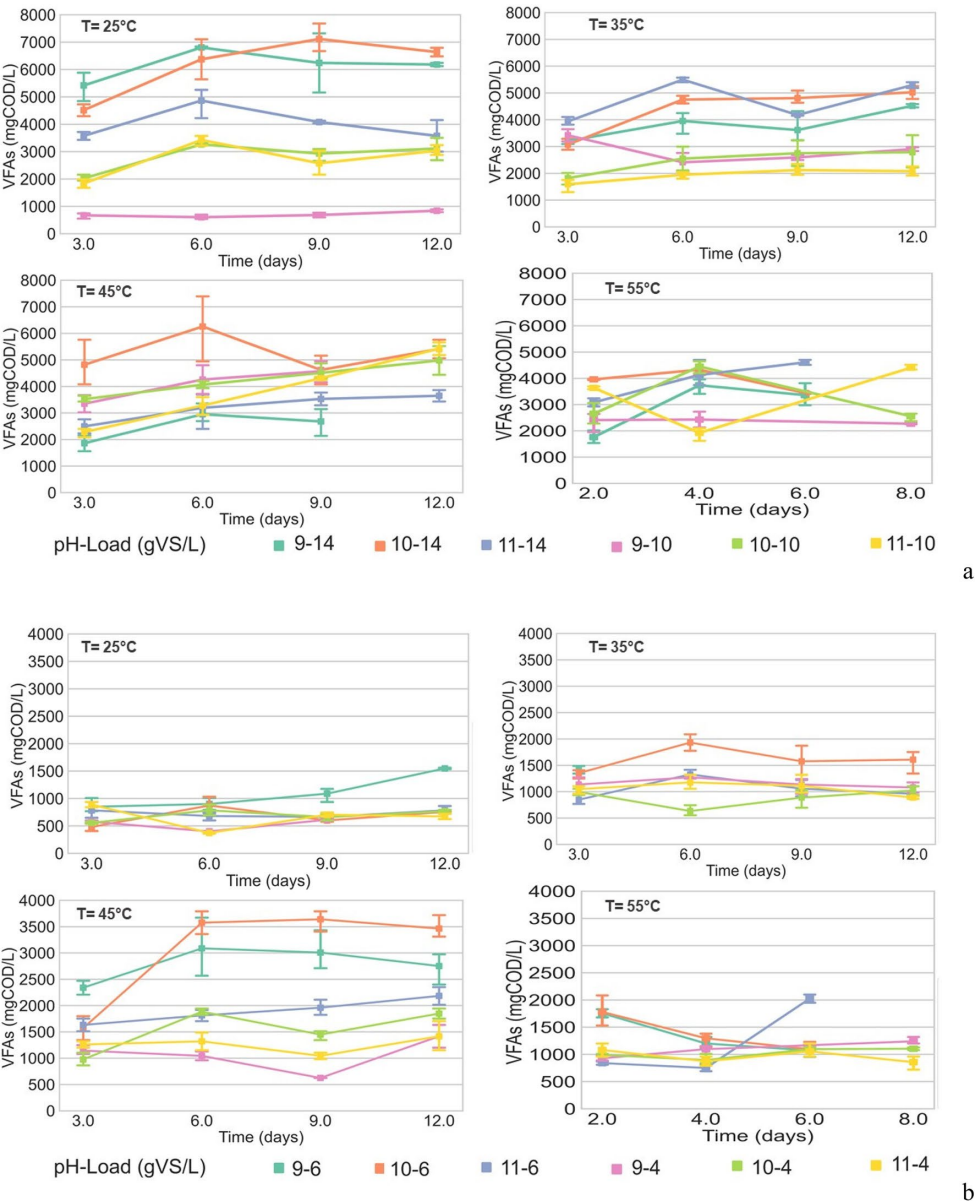
**a** VFA production in primary sludge at different temperatures, pH, and loads. **b** VFA production in digested sludge at different temperatures, pH, and loads

Figures 3a and b show the results for the different combinations. Figure 3a contains the results of the primary sludge (PS), while Fig. 3b contains those of digested sludge (DS).

The four subplots correspond to the four different temperatures, while other line colors correspond to a combination of pH and load, as shown in the legend. Error bars are sample standard deviations of triplicates.

According to the anaerobic batch fermentation results under the established conditions, the highest total VFA production using sludges from El Salitre was 7112 ± 516 mg COD/L using PS on day 9 with an OL of 14 g VS/L at a temperature of 25 °C and a pH of 10.5. The best VFA productions were obtained using PS.

The conditions of pH 9.5, with organic loading of 14gVS, 25 °C, at a retention time of 6 days, also resulted in a good production, 6840 ± 516 mgDQO/L. The highest VFA productions were always generated with the organic load of 14gVS, which suggests that this is a determinant condition in the production of VFAs. In addition, with a temperature of 25 °C, good production was generated, which is favorable for industrial-scale productions, as it could decrease the operating costs. As for the use of the 55 °C temperature, methane production was generated in a shorter time, so the experiments under this condition were short, and the production times would be shorter; however, the highest production was 4704 ± 516 mgDQO/L, with a pH of 10.5 and an organic load of 14gVS in a retention time of 6 days. These results are lower than those generated using 35 °C temperature, in which the best production was 5616 ± 516 mgDQO/L, with pH 11.5, OL of 14gVS, and a retention time of 6 days, and the best yield using 45 °C temperature, which was 6432 ± 516 mgDQO/L, on day 6, with OL of 14gVS and a pH of 10.5. According to these batch experiments, the conditions of using primary sludge with the highest organic load, pH 10.5-, and 6-day fermentation time are favorable.

In addition, OL has a direct relationship with VFA accumulation and different substrate characteristics. The results show that the PS carbon source for VFA production is at least 35% more effective. PS includes a greater diversity of compounds and physicochemical characteristics than DS, which has been treated under anaerobic conditions (Atasoy et al. 2020). Similarly, Chen et al. (2017) identified that due to the many organic compounds in sewage sludges, PS provides a good potential for VFA recovery. In contrast, the composition and properties of DS may limit its biodegradability and thus hinder VFA production due to the conversion of these compounds into biogas. Nevertheless, the results indicate that the digestate produced during anaerobic digestion of sewage sludge, contrary to expectations, might be interesting to produce volatile fatty acids under the biorefinery concept, beyond its possible use as an amendment of soils. This potential could be used to improve the economic assessment of the energetic valorization processes and define different transformation routes used as raw materials for volatile fatty acids (Cho et al. 2018; Bravo-Porras et al. 2024).

Although the total VFA yield for DS ranged from 0.09 to 0.61 (g COD/g VS), while for PS, it went from 0.04 to 0.50 (g COD/g VS), this research aimed to maximize the final VFA concentration. This substrate is a carbon source to produce polyhydroxyalkanoates (PHAs). Otherwise, the results of Ucisik and Henze (2008) reported better VFA yields using PS compared to activated sludge, (197–256 mg COD/g VS) of VFA and (11.3–25 mg COD/g VS) respectively; however, in this research, no activated sludge was used, and a better net VFA production was obtained using primary sludge.

In the acidification phase, VFA production occurs; therefore, the substrate used is critical (Begum et al. 2018). Chen et al. (2017) studied the effect of pH (7 to 10) on VFA concentration and found a maximal composition of 423 mg COD/g VS at a pH of 10. In addition, a study by Huang et al. (2018) to produce VFA using different pHs (3, 5, 7, 9, 10, and 12) indicates that optimal VFA production also occurred at pH 10. These two works, among others, coincide with the results obtained in the present study.

Regarding temperature, according to Zhou et al. (2023), this operating factor is vital for improving VFA production because it affects hydrolysis, microorganism growth, and enzymatic activity. Due to that, the production of VFAs by bioprocesses is not competitive with petroleum-based production methods because bio-based production is more expensive and less efficient. In this sense, anaerobic digestion processes must optimize VFA production yields by mesophilic temperature (Gruhn et al. 2016). The optimal temperature, however, seems to depend on the sludge type: in the results of this research, PS has higher yields at 25 °C, which is an advantage from a cost reduction point of view.

On the other hand, the residence time (RT) in anaerobic digestion depends on the operating conditions and the type of substrate (Bolaji and Dionisi 2021). In this research, the maximum time of the study was 12 days. Notwithstanding, some combinations did not make it to the final day because they produced methane. The best VFA production occurred after day 6, and the maximum values for both substrates were obtained on day 9. These results agree with Wang et al. (2014) and Shi et al. (2022). It is worth noting that the use of PS and DS as substrates in producing VFAs has yet to be studied. On the other hand, the characteristics and composition of the sludges obtained in a WWTP depend on the technology of the plant and the composition of the residual water, which is closely related to the country’s development level. Thus, this work allows for establishing the best conditions to produce value-added products and to plan strategic valorization routes for the biomass produced in these facilities (Zhang et. al 2022; Cecconet and Capodaglio 2022).

### Profile of the samples analyzed by gas chromatography

The findings on the type of VFAs produced are congruent from the point of view of the biochemical fermentation process since, according to Kumar et al. (2019), the primary expected metabolites are acetic, propionic, and butyric acid. Acetate is the direct precursor for CH4 conversion; between 65 and 95% is produced from acetic acid. Table 1 reveals the relative quantification of volatile fatty acids in some experimental treatments.

**Table 1:** Chromatographic report of the amount of VFA’s for digested sludge and primary sludge combinations

							PPM^b^							
Sludge	Initial Ph	Temp °C	OL g VS/L	RT (day)	Final pH	VFA mg COD/L ^a^	Acetic acid	Propionic acid	Iso butyric acid	Butyric acid	Iso Valeric acid	Valeric acid	Iso Caproic acid	Caproic acid
DS	10.5	35	6	12	9.76	1608 ± 0.14	837 ± 0.04	74.2 ± 0.01	133 ± 0.04	252 ± 0.04	267 ± 0.03	-	-	-
DS	9.5	45	6	6	8.32	3088 ± 0.18	1298 ± 1.05	387 ± 0.32	200 ± 0.16	184 ± 0.15	427 ± 0.36	13.9 ± 0.01	-	-
DS	10.5	45	6	9	9.94	3640 ± 0.06	920.5 ± 1.22	104.5 ± 0.08	75.5 ± 0.06	173.5 ± 0.11	202.5 ± 0.18	15 ± 0.03	-	18.5 ± 0.03
DS	10.5	45	4	9	9.54	1448 ± 0.06	616 ± 0.20	90 ± 0.01	86 ± 0.03	86 ± 0.02	168 ± 0.08	-	-	15 ± 0.02
PS	10.5	25	14	9	8.3	7112 ± 0.07	3580 ± 1.65	2180 ± 0.99	375 ± 0.17	647 ± 0.30	486 ± 0.22	226 ± 0.14	224 ± 0.17	301 ± 0.17
PS	11.5	35	14	6	9.22	5496 ± 0.02	1872 ± 0.94	1180 ± 0.67	248 ± 0.19	383 ± 0.26	525 ± 0.41	123 ± 0.09	175 ± 0.11	39 ± 0.01
PS	10.5	45	14	6	8.94	6256 ± 0.19	1594 ± 0.59	486 ± 0.17	202 ± 0.10	334 ± 0.13	381 ± 0.18	97 ± 0.5	75 ± 0.03	21 ± 0.01
PS	9.5	45	10	9	7.61	4572 ± 0.12	1582 ± 1.80	648 ± 0.78	237 ± 0.32	463 ± 0.61	392 ± 0.59	144 ± 0.19	224 ± 0.38	107 ± 0.15

^a^Average ± standard deviation. Over three samples

^b^Relative ± standard deviation

According to Wang et al. (2014), the composition of VFA was in the order of butyric, acetic, and propionic acids from highest to lowest concentration: 70% butyric, 17% acetic, 5% propionic, and 8% others. The present study obtains average concentrations: 50% acetic, 15% propionic, 10% butyric, and 6% isobutyric. Other types of VFA, such as valeric, isovaleric, propionic, and butyric acid, are better produced on high protein substrates (Garcia-Aguirre et al. 2017). The differences obtained indicate the importance of this kind of experiment when the characteristics and potential of the substrates for valorization depend on the standard of living and the climatological conditions of the place where the WWTP is located (Mosquera et al. 2020).

Generally, VFAs produced from waste streams are explained according to their organic matter content (Jankowska et al. 2015; García-Aguirre et al. 2017; Wang et al. 2014; Yin et al. 2016). Nevertheless, predicting the acid distribution based on the substrate type is difficult.

### Polynomial third-degree regression

**Fig. 4:**
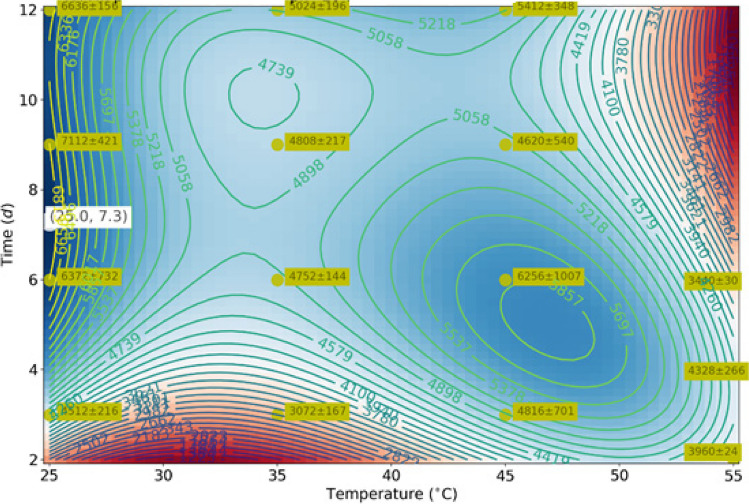
Third-degree polynomial regression treats primary sludge with an organic load of 14 gVS/L, a temperature of 25 °C, and a pH of 10.5

Bivariate third-degree polynomial regressions were performed for each treatment of organic load, pH, and type of sludge. The primary sludge with an organic load of 14 g VS/L and pH of 10.5 had the highest predicted VFA production (6975 mg COD/L), with an optimal point at a 25° C temperature and 7.3 days of reaction time and agreed with the experimental results. This result is interesting considering the possibility of scaling up the system in WWTP; using this operation temperature minimizes the process’ energetic consumption and environmental impact. The temperature of 25 °C seems to guarantee the stabilization of the system during the production of VFAs compared to the other temperatures evaluated, resulting in a higher cumulative concentration of the compounds (Gong et al. 2021). The *r*^2^ coefficient of determination of the regression for this combination was 0.83, which is reasonable, especially considering the notorious variability of some experimental points. The least-square optimization and *r*^2^ computation was performed with all empirical points having the same weight. However, several points (usually three) correspond to the same time and temperature values. The uncertainty in these experimental points implies that a very high *r*^2^ coefficient is mathematically impossible: even if the model function could pass perfectly through the average of each empirical point, the uncontrolled noise in the data would considerably lower the *r*^2^ coefficient. This substantial variability is the main reason for performing further statistical significance tests to justify the selection of this treatment as the one to be used in the pilot-scale test. Figure 4 presents the contour map of the polynomial regression for this best-performing treatment. In this figure, the experimental points are marked with their standard deviations; the optimal point is indicated in white. The other combination that presented an excellent VFA production performance was that of primary sludge at 14 g VS and pH 9.5; in 6 days, *r*^2^, in this case, was 0.92.

Although the ${r}^{2}$ coefficient is low (i.e., there is more uncertainty in the prediction) for the combination of Fig. 4, it is still highly likely that this is the best combination. To ensure this conclusion, an ANOVA test and a *t*-test were carried out so we could confirm that the optimal combination stands out despite statistical and experimental uncertainty.

An ANOVA *F*-test was performed, reducing the degrees of freedom to those available in the polynomial regression for each treatment. With this test, the *H*_0_ hypothesis of equal means was rejected, with a *p*-value too close to zero to be distinguished by computational precision (see Table 2). This result is excellent evidence that the optimal VFA production of each treatment is statistically different. Finally, the input data of this ANOVA test was obtained, estimating means and standard deviations by bootstrapping.

**Table 2:** Analysis of variance

*Origin of variations*	*Sum of squares*	*Degrees of freedom*	*Mean-squared Average of squares*	*F*	*P-value*	*Critical F value*
The sum of squares by groups	43353766	11	123701770	615.2	0.000	1.817
The sum of squares of error	67562446	336	201079			
The sum of total squares	14143970741	347				

According to the results, PS at pH 10 and load 14 predicts a similar maximum yield to pH 9 and load 14. The two best-performing treatments look relatively similar, suggesting the inclusion of the second-best one in the validation at the pilot-plant scale. This possibility was excluded by statistically comparing these two best-performing treatments through a *t*-test (pH-Organic load, 10–14 and 9–14), ensuring that the best-performing treatment stands alone (Table 3).

**Table 3:** *T*-test of the best combinations

Average of 2 (mg COD/L)	7021.46	pH-Organic load: 10–14
The standard deviation of 2	181.55	
degrees of freedom of 2	28	
Average of 1 (mg COD/L)	6855.04	pH-Organic load: 9–14
The standard deviation of 1	215.38	
degrees of freedom of 1	28	

Finally, to assess the variables’ influence on the response, a Pareto analysis was performed using the *t*-values of the coefficients of the third-degree polynomial regression of the best-performing treatment. As a result, the VFA production model is defined by Eq. (2).

**Fig. 5:**
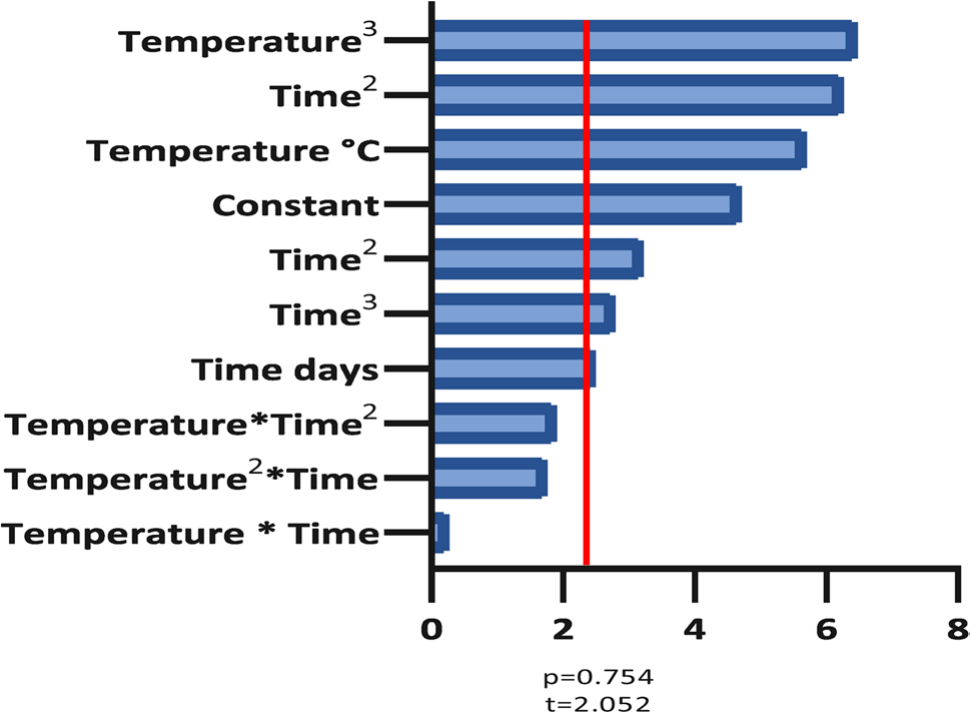
Pareto chart of the *t*-values of the regression coefficients of the best-performing treatment

If the null hypothesis is that the coefficient is 0 (i.e., no influence of the polynomial term), and the significance is 0.05, the *t*-value threshold for rejecting the null hypothesis is 2.052. In Fig. 5, it is possible to see that only three terms are below this threshold and that several third-degree coefficients have statistically significant effects. Hence, a third-degree polynomial is justified as a pertinent model for the data.

### Validation of the statistical model in the pilot plant

Table 4 shows the physicochemical characteristics of the PS used in the pilot plant experiment. These characterization results differ from those Haugaard Mikkelsen and Keiding (1999) reported. According to that study, the protein in primary sludge was 140,000 mg/L in Denmark. On the other hand, the protein in primary sludge written by García (2009) from a wastewater treatment plant in México varies between 4000 and 40,000 mg/L. From the above, it can be considered that the physicochemical characteristics of municipal sewage sludge are highly variable, depending on the conditions of each geographical area, climatic conditions, and types of treatment. Therefore, the sludge characterization can impact volatile fatty acid production.

**Table 4:** Primary sludge characterization wet-based report

Parameter	Report	Units
Total nitrogen	1861	mg/L
Fats	4214	mg/L
Proteins	11,631	mg/L
Fibers	2.93	% P/P
Carbohydrates	< 1.00	%

^b^The analysis was conducted in a specialized laboratory, ensuring the deviation is less than 10%

**Fig. 6:**
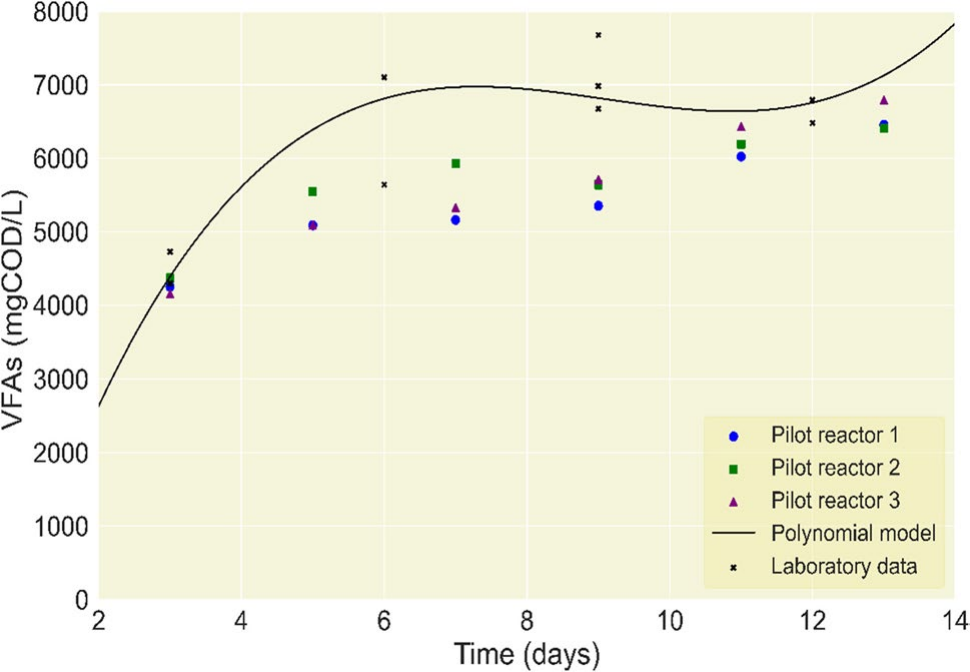
Lab-to-pilot-plant scalability results for the optimal treatment

Figure 6 shows the results obtained in the fermentation carried out for 15 days in a pilot plant with three semi-continuous reactors under the operational parameters of the best treatment according to the polynomial models (primary sludge, OL 14 g VS, pH 10, temperature 25 °C). It was possible to validate the model, as the results suggest the first optimal production on day 7 and then on day 13. However, from a technical point of view, the optimal VFA production is still on day 7, as the more days of fermentation in the reactors, the more difficult it is to control operationally. This behavior was observed during the experimental setup analysis. In the model constructed, this fact was reflected, so the curve obtained has a similar shape and trend to the results of VFA production in both scales. On the other hand, the pilot scale results are systematically lower than the laboratory scale, which can be justified by the differences in the compounds of the sludge (considering its heterogeneity) used in the two experiments. The difference may also be caused by the microorganisms being able to feed more quickly in the laboratory-scale reactors due to their size and geometry, possibly facilitating the microbial community’s mass transfer and metabolism (Singh et al. 2019).

It is important to remark that stable behavior was achieved in all three pilot plant reactors. The highest VFA production value in the pilot plant was 6792 mg COD/L, very close to the one predicted by the polynomial model of 6975 mg COD/L, with only a 2.6% difference.

## Conclusions

The production of biobased VFAs from municipal sewage sludge was developed to increase efficiency and optimize operating conditions. A statistical model was created, which determined that the best conditions for VFA production are given using primary sludge with an organic load of 14gVS, pH of 10.5, and temperature of 25 °C, and the results were validated in a pilot plant with three 5-L reactors in which there was a similar trend to that projected in the statistical model. The model is a powerful tool for evaluating and analyzing the possibility of applying a biorefinery in a full-scale WWTP.

## Supplementary Information

Below is the link to the electronic supplementary material.

Supplementary file1 (DOCX 36 KB)

## Data Availability

The authors declare that the data supporting the findings of this study are available within the paper and its Supplementary Information files. Should any raw data files be needed in another format they are available from the corresponding author upon reasonable request.
