# Supplementary material for: Statistical modeling and optimization of volatile fatty acids production by anaerobic digestion of municipal wastewater sludge
Source: Environ Sci Pollut Res Int. 2024 Aug 28;32(48):27929–41. doi: 10.1007/s11356-024-34091-2 (PMC12696022; doi:10.1007/s11356-024-34091-2)
Supplement: Supplementary file 1 — Supplementary file1 (DOCX 36 KB) [file 11356_2024_34091_MOESM1_ESM.docx]

**Table - S1.** Polynomial regression models with Primary Sludge

|  | **Primary sludge** | | | | | |
| --- | --- | --- | --- | --- | --- | --- |
|  | **pH=9.5** | | **pH=10.5** | | **pH=11.5** | |
|  | **IOL=10 gVS/L** | **IOL=14 gVS/L** | **IOL=10 gVS/L** | **IOL=14 gVS/L** | **IOL=10 gVS/L** | **IOL=14 gVS/L** |
| ***β_0_*** | 13410.8 | 6976.7 | 46042.3 | 56506.7 | 0.8 | -44684.6 |
| ***β_1_*** | -1233.1 | -362.7 | -4043.2 | -4946.5 | 10.0 | 3575.6 |
| ***β_2_*** | -1379.2 | 4106.0 | 1932.8 | 3692.5 | 1142.4 | 3287.0 |
| ***β_3_*** | 42.129 | -0.314 | 105.602 | 128.907 | 3.273 | -95.654 |
| ***β_4_*** | 30.648 | -26.225 | 31.281 | 12.703 | -53.755 | -6.315 |
| ***β_5_*** | 119.862 | -525.914 | -322.326 | -457.912 | -17.560 | -445.910 |
| ***β_6_*** | -0.422 | 0.073 | -0.857 | -1.038 | -0.035 | 0.824 |
| ***β_7_*** | -0.226 | -0.028 | -0.637 | -0.890 | 0.238 | -0.234 |
| ***β_8_*** | -0.880 | 2.368 | 0.887 | 2.497 | 3.381 | 2.041 |
| ***β_9_*** | -3.820 | 19.350 | 12.074 | 14.506 | -4.635 | 15.571 |
| **tempmax (°C)** | 45.4 | 25.0 | 47.0 | 25.0 | 47.0 | 30.3 |
| **timemax (days)** | 9.6 | 5.7 | 5.6 | 7.3 | 12.0 | 5.8 |
| **agvmax (COD/L)** | 4417 | 6836 | 4766 | 6975 | 6016 | 5589 |
| **MSE** | 169884 | 174093 | 266260 | 255240 | 245247 | 151476 |
| **r squared** | 0.915 | 0.921 | 0.699 | 0.834 | 0.769 | 0.791 |
| **p-value of F test** | 1.11E-11 | 3.74E-09 | 6.07E-05 | 4.15E-13 | 1.02E-08 | 8.19E-07 |

**Table - S2.** Polynomial Regression models with Digested Sludge

|  | **Digested Sludge** | | | | | |
| --- | --- | --- | --- | --- | --- | --- |
|  | **pH=9.5** | | **pH=10.5** | | **pH=11.5** | |
|  | **IOL=4 gVS/L** | **IOL=6 gVS/L** | **IOL=4 gVS/L** | **IOL=6 gVS/L** | **IOL=4 gVS/L** | **IOL=6 gVS/L** |
| ***β_0_*** | -16072.4 | 23927.9 | 11700.9 | 29853.6 | 1478.0 | 7370.7 |
| ***β_1_*** | 1165.4 | -1877.9 | -1001.6 | -2603.0 | -101.2 | -649.4 |
| ***β_2_*** | 1011.440 | -2384.750 | 210.295 | -406.267 | -155.836 | 289.335 |
| ***β_3_*** | -26.288 | 49.054 | 27.495 | 72.664 | 4.603 | 20.396 |
| ***β_4_*** | -31.771 | 120.074 | 2.210 | 29.064 | 2.147 | -23.468 |
| ***β_5_*** | -76.025 | 77.744 | -37.459 | 4.829 | 11.141 | 10.998 |
| ***β_6_*** | 0.193 | -0.405 | -0.235 | -0.629 | -0.052 | -0.200 |
| ***β_7_*** | 0.336 | -1.389 | -0.061 | -0.472 | -0.020 | 0.426 |
| ***β_8_*** | 0.511 | -1.657 | 0.296 | 0.636 | -0.010 | -0.194 |
| ***β_9_*** | 2.939 | -1.165 | 1.246 | -1.880 | -0.325 | -0.315 |
| **tempmax (°C)** | 47.0 | 43.1 | 47.0 | 46.2 | 45.2 | 47.0 |
| **timemax (days)** | 12.0 | 9.8 | 12.0 | 11.0 | 12.0 | 12.0 |
| **agvmax (COD/L)** | 1336 | 3246 | 1868 | 3490 | 1303 | 2389 |
| **MSE** | 20465 | 50233 | 31563 | 159053 | 25317 | 36409 |
| **r squared** | 0.763 | 0.929 | 0.750 | 0.848 | 0.647 | 0.865 |
| **p-value of F test** | 5.83E-07 | 1.10E-07 | 3.09E-05 | 3.70E-11 | 3.16E-09 | 2.58E-10 |
